# Dichlobentiazox (Pesticides)

**DOI:** 10.14252/foodsafetyfscj.D-20-00002

**Published:** 2020-03-27

**Authors:** Food Safety Commission of Japan

## Abstract

FSCJ conducted the risk assessment of a fungicide, dichlobentiazox (CAS No.957144-77-3), having benzoisothiazole and isothiazole rings, based on results from various studies.The data used in the assessment include fate in animals (rats) and in livestock (goats), fate in plants (paddy rice), residues in crops, subacute toxicity (rats, mice and dogs), chronic toxicity (dogs), carcinogenicity (rats and mice) and other relevant study results. This chemical showed none of carcinogenicity, reproductive toxicity, teratogenicity and genotoxicity. The lowest no-observed-adverse-effect level (NOAEL) obtained in all studies was 5.03 mg/kg bw per day in a two-year chronic toxicity/carcinogenicity study in rats. FSCJ specified an acceptable daily intake (ADI) of 0.05 mg/kg bw per day by applying a safety factor of 100 to the NOAEL.

## Conclusion in Brief

FSCJ conducted the risk assessment of a fungicide, dichlobentiazox (CAS No.957144-77-3), having benzoisothiazole and isothiazole rings, based on results from various studies.

The data used in the assessment include fate in animals (rats) and in livestock (goats), fate in plants (paddy rice), residues in crops, subacute toxicity (rats, mice and dogs), chronic toxicity (dogs). chronic toxicity/carcinogenicity (rats), carcinogenicity (mice), two-generation reproductive toxicity (rats), developmental toxicity (rats and rabbits), and genotoxicity.

Major adverse effects of dichlobentiazox were suppressed body weight, anemia (dogs), hyperplasia and hypertrophy of bile duct in the liver, and epithelial hypertrophy/hyperplasia of villus in the duodenum. This chemical showed none of carcinogenicity, reproductive toxicity, teratogenicity and genotoxicity.

On the basis of various studies, dichlobentiazox (parent compound only) was identified as a relevant substance for residue definition for dietary risk assessment in agricultural product.

The lowest no-observed-adverse-effect level (NOAEL) obtained in all studies was 5.03 mg/kg bw per day in a two-year chronic toxicity/carcinogenicity study in rats. FSCJ specified an acceptable daily intake (ADI) of 0.05 mg/kg bw per day by applying a safety factor of 100 to the NOAEL.

FSCJ judged it unnecessary to specify an acute reference dose (ARfD), since no adverse effects would be likely to be elicited by a single oral administration of dichlobentiazox. (table 1)

**Table 1.** *Levels relevant to toxicological evaluation of dichlobentiazox*

Species	Study	Dose (mg/kg bw/day)	NOAEL (mg/kg bw/day)	LOAEL (mg/kg bw/day)	Critical endpoints^1)^
Rat	90-day subacute toxicity study	0, 300, 900, 3 000 ppm	M: 22 F: 74	M: 65 F: 263	M: Hyaline droplet accumulation in renal tubule cortex and others F: Epithelial villus hypertrophy/hyperplasia of duodenum
		M: 0, 22, 65, 236 F: 0, 25, 74, 263			
	Two-year combined chronic toxicity /carcinogenicity study	0, 120, 550, 2 500 ppm	M: 5.03 F: 7.01	M: 23.5 F: 31.9	FM: Epithelial villus hypertrophy/hyperplasia of duodenum and others (Not carcinogenic)
		M: 0, 5.03, 23.5, 108 F: 0, 7.01, 31.9, 144			
	Two-generation reproductive toxicity study	0, 62.5, 250, 1 000	Parent M: 62.5 F: 1 000 Offspring FM: 1000	Parent M: 250 F: - Offspring FM: -	Parent M: Suppressed body weight F: No toxicity Offspring FM: No toxicity (No effect on reproduction)
	Developmental toxicity	0, 62.5, 250, 1 000	Maternal: 250 Embryo/fetus: 250	Maternal: 1 000 Embryo/fetus: 1 000	Maternal: Suppressed body weight and decreased feed consumption Embryo/fetus: Delayed ossification (absent ossification of fifth and sixth sternebrae) (Not teratogenic)
Mouse	90-day subacute toxicity study	0, 100, 450, 2 000 ppm	M: 65 F: 80	M: 315 F: 381	FM: Epithelial villus hypertrophy/hyperplasia of duodenum and others
		M: 0, 14, 65, 315 F: 0, 19, 80, 381			
	78-week carcinogenicity study	0, 50, 325, 2 000 ppm	M: 247 F: 258	M: - F: -	FM: No toxicity (Not carcinogenic)
		M: 0, 5.8, 38, 247 F: 0, 6.6, 42, 258			
Rabbit	Developmental toxicity study	0, 15, 50, 150	Maternal: 50 Embryo/fetus: 150	Maternal: 150 Embryo/fetus: -	Maternal: Decreased/suppressed body weight, decreased feed consumption and others Embryo/fetus: Not toxic (Not teratogenic)
Dog	90-day subacute toxicity study	0, 10, 70, 500	FM: 10	FM: 70	FM: Bile duct hyperplasia in liver
	One-year chronic toxicity study	0, 5, 50, 500/200	FM: 50	FM: 500/200	M: Bile duct hypertrophy in liver and others F: Decreased RBC, Ht or Hb and others
ADI			NOAEL: 5.03 SF: 100 ADI: 0.05		
The critical study for setting the ADI			Two-year combined chronic toxicity/carcinogenicity study in rats		

ADI, Acceptable daily Intake; NOAEL, No-observed-adverse-effect level; SF, Safety Factor

Lowest-observed-adverse-effect level (LOAEL) was not derived.

^1)^ The adverse effect observed at LOAEL

